# Pernicious Vomiting of Pregnancy

**Published:** 1907-12-14

**Authors:** 


### Gynæcology.

### PERNICIOUS VOMITING OF PREGNANCY.

Vomiting in the earlier months of pregnancy often gives rise to discomforts and inconvenience, but rarely leads to any symptoms which may place life in danger. When, however, vomiting is so severe that no food can be retained, the effect upon the patient may be very serious indeed. The symptoms and signs following this excessive or pernicious vomiting are rapid wasting, increased specific gravity of the urine, and passage of albumen and casts, frequency of the pulse and alkalinity of the blood, and sometimes the presence of normoblasts and megaloblasts. The causes of simple vomiting of pregnancy are exceedingly obscure, and therefore those of this pernicious form are much more so. The great variations in the degree of vomiting experienced by many patients make it abundantly clear that we are not justified in considering the vomiting of pregnancy as physiological. The various theories which have been held to account for pernicious vomiting may be grouped under three heads—namely:—

1. A reflex act due to some lesion of the pregnant uterus or pelvic organs.
2. Hysteria.
3. A toxæmia.

The first theory is the oldest, and has been much discussed. The lesions which have been described as setting up reflex vomiting are displacements of the gravid uterus, rigidity of the cervix, erosions and endometritis, peritoneal adhesions to the uterus, and unusual stretching of the uterus. Now whilst admitting that occasionally these lesions are present in cases of pernicious vomiting, it is a fact that nothing of the kind can be demonstrated in some cases. Further, treatment of these lesions sometimes fails utterly to stop vomiting.

The second theory, that pernicious vomiting is a functional neurosis is upheld chiefly by Kaltenbach. This writer points out that pernicious vomiting sometimes ceases suddenly without treatment, that hysterical stigmata may be present, and that isolation and treatment by "suggestion" sometimes produce a cure. Kaltenbach also holds that treatment of a pelvic lesion when a cure results really owes its good effect to "suggestion," and a case is mentioned by Graefe in which a cure resulted from the passage of a sound, although abortion was not produced.

The last theory, that pernicious vomiting is the result of an auto-intoxication, is the one most generally believed at the present time. The influence of toxic substances in the blood in causing eclampsia, albuminuria, and possibly chorea of pregnancy, is now so commonly believed that there is no difficulty in realising their probable relation to pernicious vomiting. The sources of toxic substances are usually held to be the alimentary canal, the fœtus and placenta, and the metabolic processes of the maternal organism in general. Toxic substances as a rule are eliminated by the kidneys, the alimentary canal, and the skin in healthy individuals, and in the majority, pregnancy has no ill effects. When, however, in a pregnant woman for some reason these toxins are not eliminated, then one of the above-mentioned diseases may result. When the cause of the non-elimination of these toxins is discovered, our knowledge of pernicious vomiting, eclampsia, and albuminuria in pregnancy will be greatly increased.

It is probable that those cases which have been cured by the treatment of a local pelvic lesion are not among the most severe. Copeman's treatment must not be forgotten — namely, the dilatation of the cervical canal and internal os without inducing abortion. This method was discovered accidentally, Copeman having determined to dilate the cervix in a case of pernicious vomiting, intending to induce abortion. The dilatation was carried out with the finger, and an attempt was made to rupture the membranes. Failing to do this the case was left alone, and curiously no further vomiting occurred. This treatment has been deliberately carried out since in many cases, with a certain amount of success. There is, of course, a considerable risk of inducing abortion if the cervix is dilated either with the finger or with metallic dilators. The method should clearly not be used until others have failed.

The most hopeful method of treatment is that based on the toxæmia theory. The first principle will be to give large rectal saline enemata, injecting slowly with the hips raised, so that the fluid may be retained. Rectal feeding must, of course, be substituted for mouth feeding, and there is no reason why milk and other nutrient substances should not be added to the enemata. It is important that the colon should be washed out daily, so that no toxic substances may accumulate. The stomach may be washed out, too, at the beginning of the treatment, and a saline purgative introduced; but, of course, there is always the difficulty in these cases of getting any purgative retained in the stomach. In very bad cases the subcutaneous infusion of saline solution may be substituted for rectal enemata, especially if there is any difficulty in the retention of the latter. Most of the drugs which have been recommended are found to be useless in serious cases, but there is one—thyroid extract—which has not yet been fully tried for pernicious vomiting. Bearing in mind the important effect which is claimed for thyroid extract in puerperal eclampsia and pre-eclamptic conditions (almost certainly depending on toxæmias) it seems reasonable that some good effect might be produced in pernicious vomiting. It may be given per rectum, if nothing can be retained in the stomach, in five-grain doses three times a day to commence with. If no evil effect is produced the dose may be increased. It may be remembered that in eclampsia, huge doses, up to forty grains, are tolerated without bad effect.

Finally, if no treatment stops the vomiting and progressive wasting, abortion must be induced, and an important word of caution should be remembered, that this operation must on no account be put off too long. Several patients have perished because they were almost moribund before abortion was induced.

